# Omega-3 Supplementation and Its Effects on Osteoarthritis

**DOI:** 10.3390/nu16111650

**Published:** 2024-05-28

**Authors:** Megan Shawl, Thangiah Geetha, Donna Burnett, Jeganathan Ramesh Babu

**Affiliations:** 1 Department of Nutritional Sciences, Auburn University, Auburn, AL 36849, USA; 2 Boshell Metabolic Diseases and Diabetes Program, Auburn University, Auburn, AL 36849, USA

**Keywords:** osteoarthritis, omega-3, supplementation, anti-inflammatory, inflammation

## Abstract

Osteoarthritis (OA) is a degenerative joint disease characterized by the destruction of the articular cartilage, resulting in a pro-inflammatory response. The progression of OA is multifactorial and is influenced by the underlying cause of inflammation, which includes but is not limited to trauma, metabolism, biology, comorbidities, and biomechanics. Although articular cartilage is the main tissue affected in osteoarthritis, the chronic inflammatory environment negatively influences the surrounding synovium, ligaments, and subchondral bone, further limiting their functional abilities and enhancing symptoms of OA. Treatment for osteoarthritis remains inconsistent due to the inability to determine the underlying mechanism of disease onset, severity of symptoms, and complicating comorbidities. In recent years, diet and nutritional supplements have gained interest regarding slowing the disease process, prevention, and treatment of OA. This is due to their anti-inflammatory properties, which result in a positive influence on pain, joint mobility, and cartilage formation. More specifically, omega-3 polyunsaturated fatty acids (PUFA) have demonstrated an influential role in the progression of OA, resulting in the reduction of cartilage destruction, inhibition of pro-inflammatory cytokine cascades, and production of oxylipins that promote anti-inflammatory pathways. The present review is focused on the assessment of evidence explaining the inflammatory processes of osteoarthritis and the influence of omega-3 supplementation to modulate the progression of osteoarthritis.

## 1. Introduction

Osteoarthritis (OA) is defined as a degenerative joint disease in which there is chronic destruction of the articular cartilage secondary to micro and macro injury, resulting in a pro-inflammatory response [1]. OA is multifactorial, and its disease progression is influenced based on the extent of inflammation and factors including but not limited to trauma, metabolism, biology, and biomechanics [2]. Articular cartilage is the smooth cartilage at joint surfaces, providing a low friction surface and enhanced transmission of loads with joint articulations. Although articular cartilage is the main tissue impacted in OA, the resultant inflammation can impact the adjacent synovium, ligaments, and subchondral bone and subsequently lead to active synovitis and systemic inflammation. For some, this results in joint pain, mainly affecting the joints of our hands, hips, knees, lumbar and cervical spine. Typically, a diagnosis is made upon imaging, X-ray, or MRI, demonstrating loss of joint space, damage to the bone, bone remodeling, and bone spurs [3].

Treatment for symptomatic OA varies widely based on the joint involved, severity of symptoms, and comorbidities. According to the Osteoarthritis Research Society International (OARSI), the first line of treatment for OA is education, land-based exercise programs, and, in some cases, weight-management programs, followed by pharmacological treatment [4]. However, due to the extent of comorbidities commonly associated with osteoarthritis, there is an increased complexity of care through the administration of medication and potential adverse responses to manage symptoms. It has been found that 67% of individuals with OA have at least one other chronic condition, which is 20% greater than someone without [5]. Upper gastrointestinal, psychological, cardiovascular, and endocrine systems were most likely to be affected, with the main comorbidities associated with OA, including stroke, peptic ulcer, and metabolic syndrome. Metabolic syndrome is described as a combination of dyslipidemia, hypertension, and diabetes. It has been classified as a state of low-grade inflammation in the body and has been shown to have a 5-fold increased chance of also having OA, likely due to the associated reductions in activity tolerance and lifestyle changes that follow [6]. In vivo and in vitro studies have shown direct negative effects of hyperglycemia, dyslipidemia, and chronic low-grade inflammation on cartilage metabolism, resulting in the progression of the OA disease process.

Most pharmacological and physical approaches do not consistently address the underlying degenerative process of the arthritic joint and are considered palliative, which begs the question: are there other treatment methods to address the disease process of OA? Diet and nutritional supplements have been gaining interest in recent years regarding disease process, prevention, and treatment. Specific to OA, diet modifications and nutritional supplements have demonstrated a positive influence on inflammation, pain, joint stiffness, and cartilage formation [7]. Omega-3 polyunsaturated fatty acids (PUFA) are recognized for having anti-inflammatory properties and have demonstrated an influential role in the inflammatory and catabolic process that contributes to the disease progression of OA [8].

The purpose of this review is to assess recent research on the efficacy of omega-3 PUFAs in modulating the progression of osteoarthritis.

## 2. Osteoarthritis

### 2.1. Prevalence

Osteoarthritis is the leading cause of disability globally, with an increase of 0.32% (95% CI, 0.28, 0.36) in aged-standardized incidence rates, or a 9% increase from 1990 to 2017 [9]. In a recent review, it was found that the number of impairments with activities of daily living was 1.12–1.35 times greater in those with OA, and typically substantially more health care consultations, medication use, and net disability days were required [10,11]. Due to the complexity of OA, many associated risk factors can lead to its presentation and risk of progression [1]. Such factors include occupation, joint injury, joint alignment, obesity, physical activity, age, gender, and genetics, to name a few. Aging is the single greatest risk factor for the development of OA in both radiographic and symptomatic presentations. According to the Framingham OA study, a large-scale, population-based, longitudinal study of knee OA, progressive disease occurs commonly in elder years and more commonly in women than men [11]. Of those studied, 27% of those aged 63 to 70 years old had radiographic evidence of knee OA, which increased to 44% after the age of 80. Hip OA, although less common than knee OA, also demonstrated an increased incidence with age from 0.7% in the 40–44 years age group to 14% in those over the age of 85 years [12]. Additionally, in women, typically after menopausal age, the risk of hand OA peaks and then plateaus, eventually reducing in older years [13]. When correlating hand, hip, and knee OA, researchers have found a correlation explaining there is a greater risk of hip and knee OA with a history of hand OA, and previous hip OA is associated with an increased risk of knee OA and vice versa.

### 2.2. Genetics

As OA affects a large majority of the population, studies have been conducted to assess if there is a correlation between genetics and OA. A genome-wide meta-analysis performed over 826,690 individuals, 177,517 of whom with OA, found 100 independently associated risk variants across 11 OA phenotypes, 52 of which are newly identified [14]. The genetic variances include genetic differences in weight-bearing versus non-weight-bearing, sex-specific, and early age-at-onset risks. There are also genetic correlations for pain, main disease symptoms, and casual genes linked to neuronal processes. Knee, hip, and hand OA are thought to be genetic, with 10–30%, 50%, and 70% risks, respectively [15]. In recent years, the growth and differentiation factor 5 gene (GDF5) has shown a consistent genetic association with OA, most notably a single nucleotide polymorphism at the 3′ untranslated region of GDF5. GDF5 is an extracellular signaling molecule in the TGF-β family, thus defining its role in immune regulation and development, maintenance, and repair of synovial joint tissues [16]. A reduction in GDF5 mRNA protein can result in a presentation similar to OA.

### 2.3. Normal Cartilage

Articular cartilage is an avascular and aneural connective tissue made up of chondrocytes that acts as a load-bearing tissue, absorbs impact, and sustains shear forces applied by articulating joints [2]. Chondrocytes are specialized, as they contain proteoglycans, collagen fibers, and a large amount of water within their extracellular matrix to provide frictionless joint articulation. Chondrocytes maintain cartilage matrix homeostasis by constant matrix synthesis and degradation. Collagen represents about 50% of the cartilage tissue, with specialized type II fibers to provide tensile stiffness and strength. Proteoglycans are made up of hyaluronic acid with multiple monomers attached laterally. These supramolecular aggregates allow for hydration and swelling abilities of the cartilage, enhancing the tissues’ ability to withstand compressive forces [17]. Water has the highest concentration, mainly located near the surface of the cartilage, with decreasing concentrations as you progress deeper towards the bone [2]. It is most responsible for the maintenance of the tissue, as well as the lubrication and nutritional status of the cartilage. Within the water component of the tissue, inorganic salts are dissolved and allow for nutrients to move throughout the layers of cartilage. This movement is necessary to provide nutrition to the deeper layers that contain more chondrocytes, which maintain the integrity of the cartilage.

### 2.4. Osteoarthritic Cartilage

Osteoarthritis has been shown to have a multitude of clinical presentations, making it difficult to determine an exact definition [18]. OA can be subcategorized into primary and secondary OA. Primary OA is idiopathic with potential influences from genetics, age, and ethnicity, whereas secondary OA includes post-traumatic, dysplastic, infectious, inflammatory, or biochemical etiologies. Furthermore, OA has demonstrated challenges to clinicians in classifying and diagnosing due to the vast symptoms and limited correlation of symptoms to radiological findings. Kellgren and Lawrence were the first to attempt radiological classification in 1957 by studying rheumatism in coal miners in Northwest England. They were able to assess intra- and inter-rater reliability by assessing eight joints, including the hand (distal interphalangeal joint, metacarpophalangeal joint, first metacarpophalangeal joint), wrist, cervical spine, lumbar spine, hips, and knees. They found that the tibiofemoral joint of the knee had the highest interobserver correlation coefficient (r = 0.83) and second highest interobserver correlation (r = 0.83) among the diarthrodial joints examined, which would eventually lead to a classification of OA-specific to the knee and would be the most widely used clinical tool in the radiographic diagnosis of OA. Using anterior-posterior knee radiographs, Kellgren and Lawrence assigned a grade of 0 to 4 to classify the severity of knee OA as in Table 1 [18].

### 2.5. Joint Tissues Involved in Osteoarthritis

The exact cause of osteoarthritis is not well understood. Many studies have examined metabolites within the serum, synovial fluid, and joint tissue to determine potential correlations to the severity of OA progression and its symptomatic presentation [19].

Serum metabolites measured in knee OA, greater than or equal to the Kellgren and Lawrence Grade 2, have demonstrated a reduction in glycine, histidine, lysophospholipid (LPC), and Branched Chain Amino Acids (BCAA) [19,20]. Glycine and histidine, after conversion to histamine, have both been associated with collagen synthesis and proliferative effects on chondrocytes, respectively, and were further reduced upon the progression of knee OA from Grade 2 to 3 and from Grade 3 to 4. Also, in this disease state, there was an observed increase in serum hypoxanthine, homocysteine, urate, tryptophan, and an increased ratio of lysophosphatidylcholine (lyosPC) to phosphatidylcholine (PC). This increased ratio of lysoPC to PC via phospholipase A2 is associated with a reduction of cartilage volume over time and is considered a prognostic biomarker of symptomatic presentation and disease progression to anti-inflammatory treatments.

Evidence has demonstrated that multiple types of immune cells, such as CD14+ monocytes and macrophages, mast cells, and CD4+ T helper cells, invade the synovium [19]. With macrophages being the most abundant immune cell in the synovium, the polarization of M2 anti-inflammatory macrophages to M1 pro-inflammatory macrophages has been demonstrated to aid in the progression of OA, as well as become a disease predictor.

Synovial fluid has demonstrated an alteration in >50 metabolites in subjects with OA as compared to a control; however, only about 5% of those metabolites that were also found in serum were correlated with synovial fluid changes [19,21]. Those metabolites include BCAA, glycine, glycerophospholipids, and creatinine. There is limited evidence in metabolite biomarkers assessed from joint tissue due to challenges in sample collection [19,22]. However, researchers found a reduction in glycine, leucine, valine, methionine, citrate, malate, and 3-hydroxybutyrate in femoral heads of adult mice as compared to its juvenile counterpart, resulting in a reduction in substrate availability required for the maintenance of chondrocyte homeostasis.

Chondrocytes rely on the metabolism of glucose via glycolysis to generate ATP [19]. However, approximately 80% of the glucose metabolized is converted to lactate to produce ATP in low oxidation states because of the varying avascular nature of cartilage. The deeper layers of chondrocytes, located in the closest proximity to the subchondral bone, are estimated to have about 1% oxygen partial pressure, as compared to about 7–10% partial pressure in the superficial layers [23]. Chondrocytes isolated from human OA joints have demonstrated a reduction in mitochondrial electrical transport chain complexes II and III and a reduction in mitochondrial membrane potential. When studied in guinea pigs with progressive OA, there is a reduction in mitochondrial reserve capacity, resulting in a reduction in ATP and mitochondrial superoxide dismutase enzymes (SOD2) production, all contributing to further mitochondrial dysfunction [24]. Additionally, acute mechanical injury from high-impact loading produces reactive oxygen species (ROS), mitochondrial swelling, changes in mitochondrial membrane polarization, impairs cellular respiratory activity, and increased proton leakage, which can lead to load-induced chondrocyte death and post-traumatic OA.

Impaired chondrocyte metabolism, as related to OA, is also involved in the limitations of energy-sensing signaling pathways [19]. AMP-activated protein kinase (AMPK) is impaired in OA chondrocytes. When impaired, AMPK will facilitate the production of pro-inflammatory factors and cartilage degradation, as well as reduce the capacity for mitochondrial biogenesis, which would otherwise be activated by the AMPK-SIRT1-PCG1α pathway and reduce the risk of development of OA. Conversely, the inflammatory processes associated with OA will activate the mammalian/mechanistic target of rapamycin (mTOR), which is a serine/threonine protein kinase that functions as a catalytic subunit for two protein complexes, mTORC1 and mTORC2. These pathways have been shown to enhance chondrocyte inflammation and apoptosis, enhancing the effects of OA.

## 3. Inflammatory Process of Osteoarthritis

Despite the variable presentation and progression of OA, the underlying pathology of inflammation remains consistent. Inflammation of the articular cartilage is due to a change in the balance of cytokines that enhances a pro-inflammatory response and, thus, the progression of osteoarthritis [25]. Pro-inflammatory cytokines, including IL-6, TNF-α, and IL-1β, will also stimulate a multitude of chemokines to cause a migration of additional pro-inflammatory cells to activate catabolic enzymes, including matrix metalloproteinases (MMPs) and a disintegrin-like and metalloproteinase with thrombospondin motif (ADAMTS).

### 3.1. Pro-Inflammatory Cytokines

Pro-inflammatory cytokine, IL-1β, is one of the most abundant in the pathogenesis of OA, as well as many other diseases [25]. It is a member of the IL-1 superfamily, including IL-1Ra (IL-1 receptor agonist). It produces its inflammatory effects by binding to the IL-1 receptor I (IL-1RI), which is a transmembrane receptor that also allows the binding of IL-1Ra and is found on many cells within the body, including chondrocytes, synoviocytes, osteoblasts, osteoclasts, and macrophages. Upon binding of IL-1β to IL-1RI, there is a cascade of signaling pathway activations that progress the pathogenesis of OA. Of these cascades, the mitogen-activated protein kinase (MAPK) signaling has been demonstrated as a predominant catabolic pathway of cartilage degradation in OA.

MAPK activates a subfamily of kinases that down-regulate aggrecan gene expression and type II collagen, contributing to the disease process of reduction in extracellular matrix production [25]. More specifically, extracellular signal-related kinases (ERKs), c-Jun *N*-terminal kinases (JNKs), and p38 MAPKs, when activated by MAPK signaling, down-regulate type II collagen, and aggrecan gene expression, and thus, a reduction in chondrocyte synthesis. ERKs are also activated by prostaglandin E2 (PGE2), nitric oxide (NO), and cycloxygenase-2 (COX-2), which are inflammatory mediators also stimulated by IL-1β. ERKs, JNKs, and p38 MAPK activate MMPs and ADAMTS, which stimulate aggrecanases and collagenases to reduce extracellular matrix synthesis and proteoglycan production, and eventually chondrocyte hypertrophy, dedifferentiation, and apoptosis. P38 MAPK and JNKs also reduce SRY-related protein-9 (SOX9), which is a master director of chondrocyte homeostasis, mediating MMP and ADAMTS, and when upregulated, is seen as a potential treatment in the early stages of OA [26].

In addition to MAPK, IL-1β has demonstrated upregulation of nuclear factor kappa-light-chain-enhancer of activated B cells (NF-ĸB), a pro-inflammatory mediator [27]. NF-ĸB upregulates COX-2, PGE2, and NO to further enhance ERK activity and reduce proteoglycan synthesis [25]. It mediates the activity of the MMP family ADAMTS-5 and ADAMTS-4, resulting in proteoglycan degradation and disruption of collagen formation while suppressing SOX9 activation, further reducing collagen synthesis as seen with JNK activation. Finally, NF-ĸB upregulates IL-8 and chemokines, CCL2 and CCL5, which, in addition to IL-6 and TNF-α (also stimulated by JNK and p38 MAPK), provide a positive feedback mechanism to IL-1β to further enhance its pro-inflammatory response.

TNF-α, a member of the tumor necrosis factor family, is a pleiotropic cytokine involved in homeostasis and disease pathology [28]. Initially, TNF-α was viewed as a cytotoxic molecule due to its activity in necrotic regression of certain tumor cells; however, due to its pleiotropic nature, it can also promote tissue regeneration, host defense, and cell survival [26]. In relation to OA, when stimulated by the pro-inflammatory cytokines, TNF-α will contribute to proteoglycan degradation and collagen disruption and inhibit collagen synthesis via cell differentiation, proliferation, and apoptosis. TNF-α is a protein secreted in two forms: membrane-bound (tmTNF-α) and the more biologically active soluble (sTNF-α) form. Tumor necrosis factor receptor 1 (TNFR1) is a membrane receptor that can be activated by both soluble and membrane-bound forms, while tumor necrosis factor receptor 2 (TNFR-2) is stimulated by membrane-bound form; each receptor has unique structural formations that result in differing signal pathways. Depending on the ligand attachment, TNFR1 can initiate one of two signaling pathways. The first, complex 1, increases the expression of pro-inflammatory genes and TNFR-1-associated death domain protein (TRADD), which in turn activates the binding of receptor-interacting protein-1 (RIP-1) and TNF receptor-associated factor-2 (TRAF-2). The binding of these two adaptor proteins results in the stimulation of NF-ĸB, MAPK and its subfamilies, and activated protein-1 (AP-1), which causes proteoglycan degradation, collagen disruption, and inhibition of collagen and proteoglycan synthesis [29]. Conversely, complex 2 is associated with programmed cell death via protein activation of Fas-associated death domain protein (FADD), procaspase 8/10, and finally, caspase 3 [30]. Last, TNFR2, when bound by TNFα, stimulates the pro-inflammatory cascade of JNK and NF-ĸB, similar to complex 1 of TNFR1, producing inhibited synthesis and degradation of proteoglycans and collagen. Due to the heightened inflammatory response resulting from TNFα and signaling cascades, there will be greater activation of pro-inflammatory cytokines and chemokines, furthering a positive feedback mechanism to progress OA.

Interleukin 6 (IL-6) is another cytokine known to contribute to the inflammatory response of OA; however, it has also been shown to participate in anti-inflammatory processes [31]. IL-6 is highly active in the immune system and expressed in chondrocytes, osteoblasts, macrophages, and adipocytes in response to IL-1β and TNF-α circulation. Its activity is initiated through binding to the IL-6 receptors on either the cell membrane (mbIL-6R) or soluble form (sIL-6R) in conjunction with gp130, a ubiquitously expressed protein that activates either trans-signaling or classic-signaling [25]. IL-6 is bound to the selective mbIL-6R and produces a classic-signaling cascade that promotes anti-inflammatory and regenerative processes [32]. This mediation of anti-inflammatory pathways is regulated by the activation of STAT3 (signal transducer and activator of transcription 3), which allows for cell proliferation and inhibition of apoptosis. Conversely, trans-signaling is active on virtually all cells due to the omnipresent pg130 and its interaction with sIL-6R, which, when in abundance, is the determining factor of trans-signaling dominance over classic-signaling. Research has demonstrated that in cases of OA with increased MMP10, ADAM10, and ADAM 17, there is proteolytic cleavage of mbIL-6R via metzincin, which causes alternative splicing of mRNA, resulting in the formation of sIL-6R. Furthermore, in patients with high-grade OA, there are significantly greater concentrations of IL-6 in synovial fluid, which is hypothesized to have a predictive value of progression of OA [33]. Utilizing baseline and 3-year follow-up serum measurements, there was an increased concentration of IL-6 and TNF-α in earlier stages of OA as compared to later stages; however, there was a poor correlation to symptom severity. Similar to the pro-inflammatory signaling cascades produced by TNF-α and IL-1β, IL-6 stimulates MAPK to enhance enzyme production of the MMP family and reduce type II collagen formation [31].

### 3.2. Pro-Inflammatory Chemokines

In addition to cytokines, chemokines have demonstrated a large role in the pathogenesis of osteoarthritis [25]. Chemokines are small molecules that produce chemotaxis, or the movement of cells within the body. These cells are known for their role in inflammation and the recruitment of monocytes and macrophages to specific locations in the body, affecting cellular responses, proliferation, and differentiation [34]. There are 4 families of chemokines that are classified based on the position of the *N*-terminal cysteine residue, including C, CC, CXC, and CX3C [25]. Most chemokines involved in osteoarthritis are in the CC (cysteine residues adjacent) or the CXC (amino acid between cysteine residues) families. These small protein ligands are activated upon binding to a G-protein coupled cell surface receptor, which shows varying levels of binding affinity but is very selective in binding subfamily receptors to their counterpart ligand (CCR to CCL). Upon binding to a chemokine-specific receptor on a leukocyte, the newly formed complex will migrate to sites of inflammation along the chemokine ligand gradient to assist in an inflammatory response. In addition to cellular motility, chemokines are also known for helping T cell differentiation and stimulation of function for adaptive immunity, as well as their pleiotropic effect enhancing the disease progression of OA, including angiogenesis [35,36].

Of the CC subfamily, CCL2, CCL3, CCL4, and CCL5 are the most pertinent to OA [36]. CCL2, also known as monocyte chemoattractant protein-1 (MCP-1), is a strong chemotactic molecule that recruits monocytes, memory T-lymphocytes, and natural killer (NK) cells and is typically found in elevated concentrations in synovial fluid of knee OA [37]. CCL2 is upregulated in chondrocytes, synovium, and fibroblasts secondary to inflammatory stimuli and continues to be elevated as the disease progresses [34]. Upon recruitment to sites of inflammation, CCR2-expressing monocytes differentiate into pro-inflammatory macrophages and drive local inflammation, playing a direct role in tissue damage. Additionally, CCL2 has been found to increase MMP-3 expression, causing proteoglycan loss and cartilaginous degradation [25].

CCL3, also known as macrophage inflammatory protein 1-alpha (MIP-1α), is produced in synovial cell types, including macrophages, neutrophils, and fibroblasts, showing enhanced expression upon inflammation [25]. In studies in murine models, it has been found that CCL3 has a specific influence on osteoclast differentiation and enhanced bone resorption, as when CCL3 was inhibited, there was a significant reduction in bone erosion when OA was present [38]. CCL4, macrophage inflammatory protein 1-beta (MIP-1β), has been defined as a unique chemokine, found to be increased in an individual’s synovial fluid with osteoarthritis, with a 28% reduction in chemotactic activity when neutralized [39]. Researchers have hypothesized that fibroblasts do not directly express CCL4 but can be stimulated to produce this chemokine upon exposure to TNF-α, IL-1β, or LPC. CCL5, regulated on activation, normal T expressed, and secreted (RANTES), facilitates leukocyte infiltration in inflamed joints of OA [40]. Researchers have demonstrated a direct correlation between CCL5 and MMP-1 and MMP-13 expression/activation via JNK and ERK protein expression, resulting in a stimulation of collagenases. Furthermore, to reduce the development or severity of OA in a mouse model, researchers found no improvements or no effect with the deficiency of CCL5 or CCR5 [38]. Finally, it should be noted that CCL2, CCL3, CCL4, and CCL5 demonstrate upregulation in human chondrocytes when treated with IL-1β [34,37,40].

Another important player in the development and progression of OA is the CXC subfamily of chemokines [25]. More specifically, CXCL8 and CXCL12. CXCL8, also known as IL-8, is active in the immune system, working as a chemoattractant of neutrophils, and is expressed by CD8+T-cells, macrophages, and monocytes, as well as fibroblasts, epithelial cells, and synoviocytes. Under pathological conditions, this protein binds to and acts on receptors CXCR1 and CXCR2, located on leukocytes, chondrocytes, osteoclasts, fibroblasts, and endothelial and epithelial cells of the nervous system [41]. Research has correlated increased levels of IL-8 with pro-inflammatory processes of osteoarthritis, contributing to the activation of MMP-13 and MMP-3 and its cascade of chondrocyte hypertrophy and differentiation [42,43]. CXCL12, also known as stromal cell-derived factor-1 (SDF-1), is another chemotactic protein that acts through the G-coupled protein receptor, CXCR4, to promote tissue integrity for repair and regrowth [25,44]. When compared to healthy controls, CXCL12 is increased 3.75-fold in the synovium of patients with OA, which in turn enhances CXCR4+ inflammatory cells into the joint space, promoting activation of MMP1, 3, 9, and 13 and thus cartilage degradation. Additionally, CXCL12 has demonstrated a role in stem cell homing and differentiation, resulting in osteophyte formation and bone erosion, two contributing factors in the progression of OA [44,45].

## 4. Anti-Inflammatory Process of Osteoarthritis

Despite an upregulation of pro-inflammatory pathways, there is a continued anti-inflammatory process that underlies an attempt to maintain homeostasis [25]. Of note, two anti-inflammatory cytokines have demonstrated a role in controlling OA progression, IL-4 and IL-10. IL-4 soluble receptor (sIL-4R) was seen to be elevated in synoviocytes and synovial fluid of patients with hand, hip, and knee OA as compared to controls, with an associated increase in CD4+T-cells [46,47]. Macrophages polarize to an M2 phenotype with anti-inflammatory properties in response to IL-4, which causes wound healing, debris clearance, and osteoclast inhibition [48]. IL-10’s pleiotropic effects have demonstrated chondroprotective processes with inhibition of MMPs, a reduction in proteoglycan degradation, extracellular matrix remodeling, and matrix homeostasis via antagonist properties on TNF-α’s caspase actions [49]. Both IL-4 and IL-10 are associated with the downregulation of IL-1β, TNF-α, and IL-6, in addition to the secretion of secondary pro-inflammatory mediators, PGE2, COX-2, and iNOS [25]. However, research has also shown that there are reductions in concentrations of IL-10 in subjects with post-traumatic knee OA with maintained TNF-α concentrations, thus allowing the progression of OA [50]. Upon disruption in the balance of anti-inflammatory and pro-inflammatory cytokines, a negative cascade of positive feedback loops promotes the pathogenesis of OA, resulting in progressive joint inflammation, cartilage degradations, and osteophyte formation.

## 5. Fatty Acids

In addition to mechanical loading, recent research has demonstrated a correlation between OA and obesity, diabetes, and metabolic syndrome [51]. Obesity increases the force on weight-bearing joints, resulting in a progressive increased risk of the development of OA. Under the conditions of a high-fat diet, there is an upregulation of fatty acid oxidation within cartilage as well as an increase in cholesterol uptake, increasing oxysterol metabolites [22]. Such metabolites will induce the expression of pro-catabolic matrix degradation enzymes. Excess intake of fat is typically accepted as an undesirable diet; however, it seems as though the quality of fat versus the quantity of fat should be taken into consideration.

Naturally occurring fatty acids can be classified into subcategories based on their structure and presence of double bonds, including saturated fatty acids (SFA, no double bond), monounsaturated fatty acids (MUFA, single double bond), and polyunsaturated fatty acids (PUFA, multiple double bonds)) [51]. There is further subclassification based on the location of the double bond in unsaturated fatty acids, with naming based on the number of carbons in the chain and the position of the first double bond about the methyl terminal (omega -ω, or n-FA) [52]. For example, all-cis-9,12,15-octadecatrienoic, also known as α-linolenic acid, ALA, or C18:3 for its 18-carbon chain length, with 3 double bonds at carbon 9, 12, and 15 counting from the methyl terminal. An additional classification refers to the number of carbon atoms within the fatty acid chain; this includes long-chain fatty acids (LCFA, ≥12 carbon atoms) and very-long-chain fatty acids (VLC-FA, ≥22 carbon atoms). Two well-known essential LCFAs that cannot be produced in the body are the previously mentioned α-linolenic acid (ALA) and linolenic acid (LA). These two are converted into VLC-PUFA by fatty acyl-CoA synthetases, Δ6- and Δ5-desaturases and their relative elongases to produce all-cis-5,8,11,14,17-eicosapentaenoic (EPA, 20:5, *n*-3), all-cis-7,10,13,16,19-docosapentaenoic (DPA, C22:5, *n*-3), all-cis-4,7,10,13,16,19-docosahexaenoic (DHA; C22:6, *n*-3), and all-cis-5,8,11,14-eicosatetraenoic (arachidonic acid, ARA, C20:4, *n*-6), which all play important roles within the human body.

### 5.1. Omega-3 and Omega-6 Fatty Acids

PUFAs are associated with positive health effects; however, *n*-3 and *n*-6 have differing roles in metabolic functions within the human body [51]. Diets with high *n*-6 PUFA are correlated with inflammation, vasoconstriction, and blood clotting, which, when in response to acute inflammation, can protect against infection, yet when in excess, can result in uncontrolled inflammation and an ideal environment for tumor growth. Conversely, *n*-3 PUFA produces an anti-inflammatory effect, altering the function of vascular and carcinogen biomarkers, including metabolic diseases, chronic inflammation, and conditions that lead to a frail musculoskeletal system. The effects of PUFA can occur as a primary event or secondary through signaling cascades utilizing bioactive signaling lipids, as well as eicosanoids. Eicosanoids are derived from metabolites of ALA and LA, including ARA, EPA, and DHA, and depending on its precursor, will determine if the eicosanoid will produce a pro- or anti-inflammatory response.

LC-PUFAs are derived from plant and animal-based foods, mainly in the form of triacylglycerols (TAG), phospholipids (PL), diacylglycerol (DAG), cholesterol esters (CE), and fat-soluble vitamins esters [52]. Positioning of the LC-PUFA on its dietary form will impact its bioavailability. For example, LC-PUFA in the form of PL (krill oil) is absorbed more efficiently than TAGs (fish oil), as there is a greater uptake of PL within the brain. Upon fatty acid oxidation, biosynthesis of LC-PUFA will occur within the endoplasmic reticulum of hepatocytes, with the process initiated by Δ6-desaturation, a rate-limiting step, adding a double bond at the 6th carbon atom from the carboxy end of LA and ALA, producing γ-linolenic acid (GLA, C18:3, *n*-6) and stearidonic acid (SDA, C18:4, *n*-3), respectively [53]. Next, a second 2-carbon elongation occurs via Δ6 specific elongase yielding dihomo-γ-linolenic acid (DGLA, C20:3, *n*-6) and eicosatetraenoic acid (ETA, C20:4, *n*-3), respectively. Last, a Δ5-desaturase adds a double bond at the 5th C—C bond and carries out one more desaturation to produce ARA (C20:4, *n*-6) from SDA and EPA (C20:5, *n*-3) from DGLA. EPA then undergoes two more elongations: tetracosanolpentaenoic acid (C24:5, *n*-3) and tetracosahexaenoic acid (THA; C24:6, *n*-3). THA is subjected to β-oxidation within the peroxisome, reducing its carbon chain by 2 atoms, achieving the final product, VLC-PUFA docosahexaenoic acid (DHA; C22:6, *n*-3). Due to the utilization of the same pool of desaturases and elongases, ALA and LA compete for bioconversion to their LC-PUFAs and are dependent upon the ratio of consumed *n*-6: *n*-3 [53]. Interestingly, the greatest formation of EPA and DHA occurs when there is a 1:1 ratio of LA: ALA, with a progressive increase in *n*-3 status upon greater ingestion of ALA [54].

Upon synthesis, ARA, EPA, and DHA are esterified and stored in PL or neutral glycerides and, when necessary, can be remobilized by phospholipase A2 to form eicosanoids [52]. ARA, derived from *n*-6 PUFAs, are converted to pro-inflammatory cytokines, prostaglandins (PGE2), and cyclooxygenase-2 (COX-2). By contrast, *n*-3 PUFAs are metabolized to anti-inflammatory series 3 prostaglandins, series 5 leukotrienes, E-series resolvins, D-series resolvins, protectins, and maresins. Resolvins, protectins, and maresins are all metabolites of oxylipins classified as specialized pro-resolving lipid modulators (SPM) [55].

### 5.2. Anti-Inflammatory Processes of Omega-3 PUFAs

SPMs reduce pro-inflammatory immune cells, increase the production of anti-inflammatory mediators, and counter-regulate pro-inflammatory mediators [56]. At the cellular level, this occurs through macrophage phagocytosis of pathogens, apoptotic cells, and cellular debris, and limits the infiltration of neutrophils and reduces chemokine and cytokine mediators to promote tissue repair and regeneration. In a study with adults diagnosed with knee OA, SPMs were administered for 12 weeks in the form of 500 mg soft gels; pain was significantly reduced at both 8- and 12-week follow-ups (*p* = 0.039 and *p* = 0.031, respectively), as well as improvements in self-reported quality of life.

Resolvins, including the E-series from EPA (RvE1, RvE4, 18S-RvE1) and D-series from DHA (RvD1 to RvD6), act as agonists in peak periods of inflammation [57]. They are metabolically catalyzed by lipoxygenases (LOX), cytochrome P450, and cyclooxygenases (COX). Resolvins combine with G-protein-coupled receptors expressed in monocyte-macrophages, lymphocytes, endothelial cells, vascular smooth muscle cells, and neutrophils, all of which are cell and organ-specific. Resolvin cellular targets depend on a unique receptor; however, there can be common target cells for different resolvin subtypes, yet with different imposing actions. RvE1 has been shown to regulate CD18 in neutrophils, reduce the phosphorylation of MAPK, and inhibit superoxide production and their migration between epithelial and endothelial cells. Additionally, RvD2 inhibits neutrophil chemotaxis to reduce migration and thus reduces the progression of inflammation. Through multiple pathways, resolvins also mediate the macrophage response to inflammation. This occurs through the conversion of M1, pro-inflammatory macrophages, to M2, anti-inflammatory macrophages, downregulation of monocyte conversion, modulation of macrophage polarization, and manipulation of macrophage targeting IL-10 and 5-LOX. Leukocytes, a key inflammatory contributor resulting in tissue damage, are regulated by resolvins by reducing the expression of CD4+T-cells, facilitating cell migration of NK to remove eosinophilic granulocytes, and reducing the secretion of TNF-α and IL-6 cells. In mouse models with osteoarthritis, researchers found RvD1 to have dampening effects on the pro-inflammatory activity of synovial macrophages through recruitment of a greater concentration of M2 cells, and it was able to target and reduce osteophyte formation with an overall analgesic effect [58]. Research has also shown that RvD1 reduces the NLRP3 pathway through the inhibition of caspase-1 signaling, which would otherwise lead to programmed cell death in chondrocytes of arthritic joints [59]. Furthermore, in humans undergoing total knee arthroplasty, synovium, and fibroblast-like synoviocytes were obtained and treated with concentrations of RvD1, finding a reduction in MMP-13 and IL-1β secretion, suggesting a possible treatment method for late-stage OA [60]. Similarly, research has synthesized an imidazole-derived RvD1 analog, finding greater antioxidant actions than RvD1 derived from DHA, including scavenging ROS, thus protecting hyaluronic acid from degradation [61].

Protectin D is derived from double lipooxygenation of DHA [55,62]. Protectin DX (PDX) is the most widely evaluated member of the protectin family, demonstrating involvement in anti-inflammation pathways in both chronic and acute inflammatory processes through its effects on AMPK signaling. When studied in vivo and in vitro, Rat models with knee OA, preincubated with PDX and then treated with IL-1β, demonstrated a suppression in inflammation in chondrocytes [62]. There was a dose-dependent reduction in inhibition of type II collagen degradation as well as an increase in AMPK phosphorylation. Activation of AMPK through phosphorylation has been shown to reduce NF-ĸB nuclear translocation, thus reducing its pro-inflammatory response, seen in this study by a reduction in expression of MMP-3, MMP-13, ADAMTS4, iNOS, COX-2, NO, PGE2, and absent phosphorylation of NF-ĸB [62,63].

Maresin-1, another metabolite of DHA, has also been shown to contribute to an anti-inflammatory response in osteoarthritis, as well as pain hypersensitivity and postoperative neuroinflammation [52,64]. It is produced by 12/15-lipoxygenase found in M2 macrophages and acts by restricting neutrophil infiltration, downregulating inflammatory factors, enhancing phagocytosis, and promoting regeneration [64]. Using a rat model with severe tibiofemoral cartilage damage through monosodium iodoacetate-induced (MIA) OA, researchers found a positive response to the administration of maresin-1 when compared to OA without maresin-1 treatment and a control group. There was a significant reduction in cartilage damage with an improvement in type II collagen when compared to the untreated and control groups. MMP13 significantly decreased in the maresin-1 treatment group through inhibition of NF-ĸB and activation of the PI3K/Akt pathway, which plays an important role in cell proliferation and survival [64,65].

## 6. Supplementation

Over the years, pharmacological treatments have been assessed to treat symptoms of osteoarthritis, yet with variable success [66]. Through suppression of COX and PGEs, NSAIDs are often used, yet with limited symptom improvement and frequently reported side effects. Acetaminophen and opioids have been prescribed; however, there is always the question of whether the risk of an unfavorable response outweighs the advantage of potential pain control. With the limited success of pharmacological therapies, there has been a growing interest in nutrition-based interventions in the treatment of OA, specifically with the use of omega-3 PUFAs [8]. Omega-3 PUFAs are typically found in fatty fish, seafood, and dietary supplements; however, traditional Western diets typically have a greater concentration of omega-6 fatty acids, which compete for metabolic enzymes, potentially reducing the positive effects of omega-3 PUFA [67].

### 6.1. Fatty Acid Composition: Ratio of Omega-6 to Omega-3

The fatty acid composition of cell membranes is altered based on the concentration of fatty acids ingested due to their role in the formation of phospholipid bilayers, lipid-mediated signaling, and gene expression [8]. Additionally, a high-fat diet is correlated with weight changes and thus altered loading response of lower extremity joints and a greater inflammatory response within joint-specific tissues [68]. Furthermore, when osteoarthritic chondrocytes are cultured with either linoleic acid (*n*-6 PUFA), oleic acid (MUFA), or palmitic acid (SFA), only linoleic acid was found to produce a pro-inflammatory response [69]. All fatty acids were easily absorbed by the chondrocyte, resulting in a higher concentration of intracellular lipids; however, while linoleic acid enhanced PGE2 via TNF-α, both oleic and palmitic acid inhibited MMP1 gene expression and GAG release, demonstrating altered influence of disease progression depending on the level of specific fatty acids. However, when supplementation of conjugated linoleic acid (CLA) was paired with EPA, it was found to have the greatest effect on reducing concentrations of PGE2 in human osteoarthritic cartilage when compared to combinations of CLA with other *n*-6 PUFAs [70].

Looking more specifically at a ratio of *n*-6 to *n*-3, multiple variations have been evaluated to determine if a pro-inflammatory or anti-inflammatory response dominates. When assessed in a rat model with osteoarthritis, rats fed with a higher ratio of *n*-6/*n*-3 resulted in a greater concentration of *n*-6 fatty acids within the trabecular bone [71]. A ratio of 1:1, *n*-6/*n*-3, demonstrated a significant reduction in MMP-13 expression, up to a 6:1 ratio, comparable to the control group, but no effect was seen at a ratio of 10:1. Similarly, paw swelling significantly increased in the 365:1 treatment group (*p* < 0.01), while decreased in the 1:1 and 2:1 (*p* < 0.01). In a human model, without supplementation, *n*-6/*n*-3 ratios were examined in subjects with knee OA about pain and functional abilities, finding that those with a greater ratio reported greater pain and functional limitations as compared to a lower ratio (*p* < 0.04) [72].

Utilizing this information, research has assessed the correlations between dietary intake of total fat, saturated fatty acids, MUFA, and PUFA as they relate to the specific cellular progression of knee OA [68]. A positive association with the progression of a disease state was found with increased total fat and saturated fat, yet a negative relationship with MUFA and PUFA. More specifically, there was a significant reduction in joint space width in those with greater intakes of saturated fatty acids and an associated progressive reduction of joint space with increased total fat intake over 48 months (*p* = 0.02). Conversely, dietary fat intakes with a higher ratio of MUFA and PUFA resulted in maintenance of joint space width or a reduction in joint space loss as compared to total fat and saturated fat.

### 6.2. Omega-3 Supplementation and Synovial Fluid

Looking a little deeper into the joint, synovial fluid has also been examined to assess the effects of fatty acid manifestations as related to the progression of joint inflammation [73]. Samples of synovial fluid were collected from shoulders and knees in individuals undergoing surgery for end-stage OA. Samples of the knee displayed lower proportions of linoleic acid (18:2, *n*-6), DHA (22:6, *n*-3), and total *n*-6 PUFAs when compared to a control. However, this was not seen in samples collected from the shoulder, potentially due to the absent altered joint response to weight-bearing forces and its associated pathological cascade. This was confirmed in an additional study assessing synovial fluid profiles of the arthritic knee in subjects participating in the Multicenter Osteoarthritis Study (MOST) [74]. This study demonstrated a positive association between *n*-6 PUFA and synovitis, yet an inverse relationship between *n*-3 PUFAs and patellofemoral cartilage loss, but not tibiofemoral cartilage loss.

### 6.3. Omega-3 Supplementation and Chondrocytes

Utilizing diet, researchers have compared a traditional Western diet with an *n*-6/*n*-3 ratio of 22:1 to a high *n*-3 diet, 1.5:1, to determine the influence of dietary supplementation on OA-prone chondrocytes [75]. In a guinea pig model, a high *n*-3 diet allowed for a reduction in OA disease progression (*p* = 0.048), achieving histological profiles similar to those who are OA resistant. There was a significant reduction in GAG content (*p* = 0.003), a reduction in denatured type II collagen, and a significant reduction in active MMP-2 levels (*p* < 0.002). Supplementation with Antarctic krill oil has also been used to achieve a diet high in omega-3 or a low *n*-6/*n*-3 ratio [76]. A diet with an *n*-6/*n*-3 ratio from 1:1 to 6:1 demonstrated significant improvement in cartilage structure and inhibition of cartilage polysaccharide loss in mice with osteoarthritis. It was demonstrated that this ratio inhibited the NF-ĸB signaling pathways and enhanced PPARγ, which inhibits inflammation and cartilage degradation, concluding that dietary intervention could be a potential treatment for OA. In addition to krill oil, the *n*-6/*n*-3 ratio of linseed oil (1:3.85), soybean oil (9.15:1), and peanut oil (372.73:1) have also been tested to determine the influence on OA [77]. After 12 weeks of supplementation with linseed and soybean oil, mice with OA had increased cartilage thickness and decreased TNF-α in both serum and chondrocytes, with no effects seen in the peanut oil group. There were also findings of inhibited NF-ĸB pathway and its resultant pro-inflammatory enzymes MMP-13 and ADAMTS-5. A human model with IL-1β induced chondrocytes, treated with DHA, demonstrated enhanced chondrocyte proliferation and reduced apoptosis, resulting in thicker cartilage, increased autophagy, and reduced apoptosis through inhibition of JNK and p38 signaling pathways [78].

### 6.4. Omega-3 Supplementation and Pain, Stiffness, and Physical Function

Based on former findings in animal models, researchers tested the response to fish oil on human subjects with knee OA, finding limited evidence in the short term [79]. In a randomized, double-blind trial, subjects with knee OA were provided with either a high-dose supplement (4.5 g *n*-3) 15 mL/d or a low-dose (blend of fish and sunola oil, 1:9 ratio with 0.45 g omega-3) 15 mL/d. Improvements were found in both groups; however, no changes in cartilage volume were achieved, and interestingly, the low-dose group had a greater reduction in pain and improved function at the 2-year follow-up. These researchers noted that this could be due to a placebo effect; however, there was no control group to compare baseline values, suggesting further research to determine human responses. In more recent research, human subjects with mild to moderate knee OA supplementing with krill oil demonstrated moderate improvements in knee pain, stiffness, and physical function [80]. Over 6 months, participants were randomly assigned to either a placebo group (mixed vegetable oil) or an intervention group (4 g krill oil/d—0.60 g EPA, 0.28 g DHA). Those with omega-3 supplementation demonstrated an increased omega-3 index (*p* < 0.001), improvements in knee stiffness and physical function (*p* < 0.05), and improvements in pain in both groups.

New Zealand Green Lippped Musssel (GLP) has demonstrated a promising influence on treatment for osteoarthritis due to its anti-inflammatory properties, attributed to its make-up of long-chain omega-3 fatty acids [81]. When GLP is administered in supplemental form (600 mg/d) in subjects with moderate to severe hip OA, there was a reduction in joint stiffness (*p* = 0.046) and pain medication use (*p* = 0.001) after 12 weeks [82]. However, despite these significant benefits, this did not achieve a clinical benefit, as there were no significant differences in pain or quality of life when compared to individuals in the control group.

The use of gelatin hydrogels has also been evaluated as they have demonstrated the ability to contain a physiologically active substance with gradual release over 3 weeks [83]. Researchers assessed the effect of incorporating EPA into the gelatin hydrogels to determine a potential beneficial response in a mouse model with OA. When compared against direct injection of EPA, the hydrogels provided a significantly lower histological profile of MMP-3, MMP-13, and IL-1β, as well as a significantly lower presence of macrophages (*p* = 0.004). Additionally, this study demonstrated that the gelatin hydrogels containing EPA had more potent effects than a single EPA injection and that the EPA in the hydrogels was fully released within 4 weeks with effects maintained for approximately 8 weeks, suggesting a potential approach for OA treatment.

### 6.5. Omega-3 Supplementation for OA with Comorbidities

Omega-3 PUFAs have also been shown to have cardioprotective effects, specifically in those with coronary artery disease (CAD) [84]. Aerobic exercise and capacity are inversely related to cardiovascular morbidity, which can be perpetuated by pain and limited physical function from osteoarthritis. Taking into consideration omega-3’s cardioprotective and symptom-mediating effects, researchers have assessed whether supplementation with EPA and DHA could maintain physical function and improve tolerance to physical activity in subjects with CAD and limited by osteoarthritis symptoms [85]. Participants were split into the experimental group, supplementing with Lovaza (465 mg EPA, 375 mg DHA) 4× daily (total of 3.36 g omega-3) or no supplementation for one year. The treatment group found a significant reduction in triglyceride levels (*p* < 0.001), hs-CRP levels (*p* = 0.023), white blood cell count (*p* = 0.029), and neutrophil count (*p* = 0.007). At the one-year follow-up, the control group experienced a significant increase in pain and stiffness (*p* = 0.002 and *p*-0.001, respectively) and worsening physical function (*p* < 0.001) compared to no change in the intervention group. Following the study, those who partook in supplementation had a 47-minute increase in total minutes of physical activity (*p* = 0.044), which was significantly higher when compared to the control group (*p* = 0.028). Furthermore, 4 subjects in the control group underwent total hip or knee surgery due to the severity of OA symptoms, whereas no subjects in the treatment group required surgery. This trend was similar at the 30-month follow-up, resulting in surgical intervention for 11 members of the control group and 1 member of the Lovaza group. This study concluded that high doses of omega-3 supplementation were able to preserve physical function and reduce musculoskeletal events without adverse effects.

### 6.6. Omega-3 Supplementation and Its Indirect Effects on OA

Despite the varying evidence for omega-3 supplementations’ direct effect on osteoarthritis progression and symptoms, researchers have demonstrated the indirect effects of omega-3 supplements on muscle recovery following exercise. A common non-pharmacological treatment for osteoarthritis is exercised to improve or maintain joint mobility flexibility and promote muscle power and function, thus reducing unnecessary loads from the joint [86]. Studies have demonstrated a reduction in the isokinetic force of the hip muscle in the setting of knee OA, with a direct correlation to pain and self-reported functional ability [87,88]. When tested in community-dwelling adults between the ages of 60- and 85 years old, fish oil-derived omega-3 supplementation allowed for improvements in quadriceps muscle volume, hand grip strength, and 1RM strength (all *p* < 0.05). This reduction in age-related muscle mass decline is imperative to maintaining functional independence and physical activity levels, thus reducing the risk of comorbidities. Similarly, omega-3 supplementation has demonstrated a positive influence on the onset of delayed onset muscle soreness (DOMS) following excessive eccentric contractions (ECC). DOMS results in a reduction of muscle power, decreased joint range of motion, and muscle swelling, peaking 1–3 days and lasting up to 7 days following ECC and/or increased exercise [89,90]. Supplementation with 600 mg EPA and 260 mg DHA, 8 weeks before initiating exercise and continued 5 days following the intervention, demonstrated improvements in joint range of motion (*p* < 0.05), increased maximal voluntary contraction (*p* < 0.05), and reduction in reported muscle soreness (*p* < 0.05). This study was repeated with a 4-week duration, finding similar results in maintained joint range of motion (*p* < 0.050), yet no differences in the other outcome measures when compared to the control group [91]. However, researchers found a significant reduction in creatine kinase levels 3 days following ECC, demonstrating a reduction in muscle tissue damage. By enhancing recovery following exercise, individuals in an OA or healthy state will be able to promote and maintain mobility and physical activity that would further reduce the risk of the progression of comorbidities related to osteoarthritis.

### 6.7. Omega-3 Supplementation in Combination with Other Nutraceuticals in OA

Multiple supplemental and nutritional products have been evaluated to reduce the physical and psychological impact of OA [92]. Moreover, researchers have assessed the positive response to such nutraceuticals in combination with omega-3 supplementation. A food supplement, Phytaligic (fish oil, vitamin E, and Urtica doica), was created and evaluated for its effectiveness in patients with hip or knee OA as compared to a control with regular use of NSAIDs [93]. After 3 months of supplementation, there was a significant decrease between groups in the use of NSAIDs (*p* < 0.001) and WOMAC scores for pain, stiffness, and function (*p* < 0.001), concluding a positive response to osteoarthritis symptoms and reduced need for analgesics.

Curcumin is a Neutraceutical agent that has been studied for the treatment of osteoarthritis due to its anti-inflammatory properties [94]. Research has evaluated if omega-3 and/or curcumin could be used as a substitute treatment method for OA, and fish oil was found to be superior to curcumin both alone and in combination [95]. This study, in particular, administered fish oil made up of 2000 mg/d DHA, 400 mg/d EPA, and 160 mg curcumin, or both for 16 weeks in obese individuals with OA-related chronic pain. Researchers found that the fish oil supplement significantly reduced OA-specific pain (*p* = 0.002) and burden (*p* = 0.015) associated with changes in microvascular elasticity, CRP inflammatory markers, and wellbeing, compared to a control, and no change in the curcumin group.

Supplementation with glucosamine sulfate has been established as a common treatment for osteoarthritis despite its variable results in research [96]. Similar to omega-3 PUFAs, glucosamine functions as a substrate for the biosynthesis of chondroitin sulfate, which is required for the development and maintenance of cartilage and extracellular matrix [97]. Researchers have assessed if supplementation of glucosamine alone or in combination with omega-3 influences symptoms of OA, finding a greater improvement in combination supplementation (*p* = 0.004) in reducing morning stiffness of hips and knees. These findings were allocated to the fact that the omega-3 content assisted in the reduction of cartilage degradation by controlling the inflammatory response, resulting in a reduction of swelling and, thus, the symptom of stiffness.

Recent research has highlighted the role of vitamin D influence on OA due to its role in muscle strength and bone resorption, with similar anti-inflammatory properties as omega-3 PUFAs [98]. Studies have demonstrated a correlation between low vitamin D levels and the progression of OA; however, results remain conflicted. A large double-blind, placebo-controlled trial was conducted over 5 years to test the response of vitamin D (cholecalciferol 2000 IU/day) and omega-3 (1 g/day, 840 mg EPA and DHA 1.3:1 ratio) supplementation on chronic knee pain secondary to OA. No significant differences were observed between either vitamin D or omega-3 and their respective control groups on symptoms of OA. This study, however, did not consider radiographic findings, which could have demonstrated changes in the quality of joint space despite limited improvements in symptoms.

### 6.8. Diet Rich in Omega-3 and Osteoarthritis

Obesity is a modifiable risk factor for osteoarthritis symptoms [99]. Clinical guidelines for knee OA recommend diet and exercise as a safe and effective approach to relieve pain and improve function. However, weight loss can be difficult when attempting to utilize exercise secondary to pain and disability related to OA progression. Simply administering a calorie-restricted diet, no less than 1100 kcal/day for women and 1200 kcal/day for men, with appropriate calorie distribution and gentle exercise, resulted in a significant, yet small, reduction in knee pain over 18 months.

In addition to reduced biomechanical forces with weight loss, losing weight is also associated with a reduction in pain-associated inflammation [100]. This is further supported when administering a diet rich in food sources containing anti-inflammatory compounds, including food sources rich in omega-3 PUFAs. Research has evaluated an anti-inflammatory diet in comparison to a calorie-restricted diet in women over the age of 40 referred to physical therapy for knee pain related to OA. The low-calorie diet requires the consumption of 500 kcal/day less energy than the total energy expenditure of each individual. This intervention was designed to consume fat less than 30% of calories, carbohydrates 55–60%, and protein 10–15%. The anti-inflammatory diet relied on fruit and vegetables in large quantities, protein from plants (legumes, soy, nuts, seeds) and fish sources (encouraged to supplement with omega-3 in addition to normal intake), and plant sources of fat, including walnuts and flaxseed rich in alpha-linoleic acid. After 2 months of intervention, there was a reduction in pain in both groups (*p* < 0.001), with a significant reduction in the anti-inflammatory diet group when compared to the calorie-restricted diet (*p* < 0.001). Only the anti-inflammatory group demonstrated significant increases in quality-of-life subscales of general health (*p* < 0.001), pain (*p* = 0.002), emotional wellbeing (*p* = 0.006), vitality (*p* = 0.031), physical function (*p* < 0.001), and a significant reduction in depression and anxiety (*p* = 0.003 and *p* = 0.011, respectively). This study also found that the weight loss was three times greater in the anti-inflammatory group, which was attributed to the use of plant proteins and vegetables, reduction in trans fatty acids, and use of omega-3-rich foods.

High-fat diets, consisting of greater concentrations of trans and saturated fats, have demonstrated increased risk factors and pain in individuals with OA [101]. Conversely, the Mediterranean diet emphasizes fruit and vegetables, whole grains, and healthy fats (omega-3 sources of olive oil, nuts, fatty fish, and low-fat dairy) and has been shown to reduce the prevalence of OA in some individuals [102]. A total of 129 patients with knee OA between the ages of 45 and 70 were included in a study evaluating the effectiveness of a Mediterranean diet versus a low-fat diet on biometrics, pain, physical function, and stiffness over 12 weeks. The Mediterranean diet consisted of 35%, 50%, and 15% calories from fats, carbohydrates, and proteins, 27–37 g of fiber per day, and at least one serving of nuts and legumes per day. The low-fat diet contained 20%, 65%, and 15% of total daily calories from fats, carbohydrates, and protein, with no additional dietary advice. Both groups were compared against a control group, which was asked not to change its dietary patterns. This study found weight and waist circumference to be significantly reduced in the two intervention groups (*p* < 0.001), as well as pain, stiffness, and physical function. However, there was only a significant difference in pain in the Mediterranean diet compared to the low-fat diet (*p* = 0.02). The onset of knee pain in standing improved by 100% in the Mediterranean diet group (*p* = 0.01), and morning stiffness decreased by 81% after 12 weeks. The authors concluded that the dietary components of the Mediterranean diet are effective in pain modulation of knee osteoarthritis.

As explained, diet and nutrition have the potential to play an important role in pain control and functional activity tolerance in osteoarthritis. The high concentrations of omega 3, an anti-inflammatory source, within the Mediterranean diet have demonstrated a positive influence on symptom management in OA; however, there are limited studies on its influence on serum biomarkers of inflammation [103]. Dyer, Davidson, Marcora, and Mauger recruited 99 volunteers with a clinical diagnosis of OA to participate in a 16-week dietary intervention to assess the systemic response of a Mediterranean diet. Subjects were randomized into either a diet group, provided with nutritional information and dietary advice consistent with a Mediterranean diet, or into a control group, provided without additional information, and blood samples were taken pre- and post-intervention. This study found all members were similar in baseline biomarker measurements, yet, at post-treatment analysis, there was a significant reduction in pro-inflammatory IL-1α (*p* = 0.019) and a non-significant reduction in serum cartilage oligomeric matrix protein (a marker for cartilage degradation, *p* = 0.057) in the diet group, and no change seen in the control group. However, this study did not find a significant change in additional biomarkers, including the interleukin family, IFN-γ, TNF-α, and EGF. Despite the lack of significant response, it has been explained that relatively small changes in biomarkers have been shown to precede radiological evidence of changes in the joint [104]. In addition to biomarkers, these researchers demonstrated significant improvements in hip and knee ROM (knee flexion *p* = 0.072, and hip rotation *p* = 0.010) following the diet intervention when compared to the control [103]. These results suggest potential benefits through dietary intervention, yet they should be further studied in the long term to determine potential further or prolonged benefits.

## 7. Dietary Sources of Omega-3

**Figure 1:**
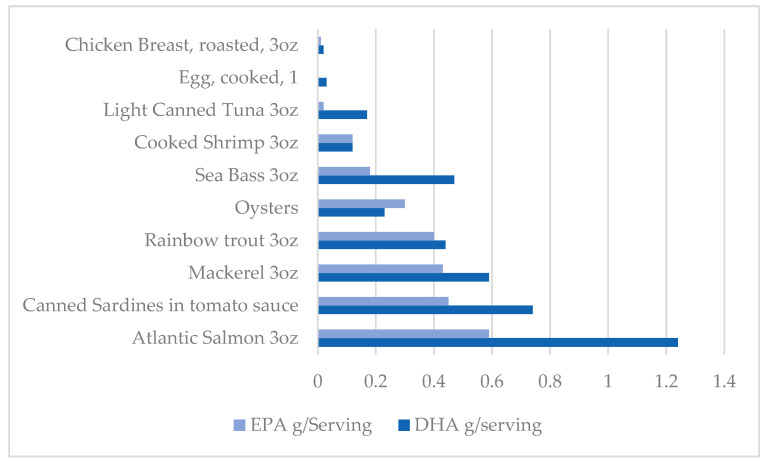
Dietary sources of Omega 3 PUFA.

According to the National Institute of Health (NIH), adequate intakes of omega-3 PUFAs in those older than the age of 19 are 1.6 g/d in males and 1.1 g/d in females, with increased requirements for females during pregnancy and lactation [105,106]. In addition to food sources seen in Figure 1, omega-3s are available in dietary supplement form [105]. Doses have a wide variety; however, supplements typically provide about 1000 mg of fish oil, which contains 180 mg of EPA and 120 mg of DHA. Some supplements are provided in marine oil form, which is also a rich source of fat-soluble vitamins [107]. In particular, the liver oil of cod, haddock, halibut, shark, whales, and tuna are typically used to produce vitamins A and D, containing approximately 1000 and 10 IU/g, respectively. Omega-3s are presented in many different forms in supplements, including natural triglycerides found in marine oils, free fatty acids, ethyl esters, re-esterified triglycerides, and phospholipids. Supplements in the form of re-esterified triglycerides, natural triglycerides, and free fatty acids have been reported to have greater bioavailability than ethyl esters; however, consumption of any form of the supplement will enhance plasma EPA and DHA levels [105].

The Institute of Medicine (IOM) developed the acceptable daily macronutrient range (ADMR) based on potential adverse effects [105]. The AMDR for omega-3 as ALA is established as 0.6–1.2% of energy for children and adults, with 10% of that intake safe to be consumed as EPA and/or DHA. The IOM has not established the UL for omega-3 but has explained that high dosages of EPA and/or DHA (900 mg/d and 600 mg/d, respectively) for several weeks or years have the potential to reduce immune function due to suppression of the inflammatory response, increase the bleeding time by a reduction in platelet aggregation, and slightly increase the risk of atrial fibrillation in those with CVD or at high risk of CVD. The FDA has concluded that dietary supplements providing ≤5 g/d of EPA or DHA are considered safe if used as recommended [105,108]. Looking at the studies explained above, most utilized a supplement value under 5 g/day, with only a few that exceeded this amount; however, they were performed in short duration with no adverse responses reported. Further evaluation of current research on omega 3’s influence on OA symptom management would be beneficial in determining the optimal dosage of supplementation and allowing improvements in future research.

## 8. Limitations

Limitations in current evidence for omega-3 PUFA supplementation include many factors. The dosing of supplementation, as well as the ratio of *n*-6/*n*-3, or DHA to EPA, remains unstandardized, bringing about variable responses. The amount of research utilizing human subjects is minimal. Most studies are performed on animal models, and despite positive results, they have yet to have the same profound response in human tissues. This is likely secondary to the complexity of variables in humans, including the onset and state of progression of the osteoarthritic joint, the subject’s dietary habits and comorbid status, and the ability of a human to produce compensatory mechanisms at a cellular level. Additionally, the type of supplementation used, plant versus animal sources, will affect the bioavailability and thus influence the potential outcomes.

## 9. Conclusions

Osteoarthritis is characterized by alterations in molecular, anatomical, and physiological processes, with influencing factors of progression from trauma, metabolism, biology, and biomechanics [86]. The underlying etiology of osteoarthritis is not well understood; however, the common theme of inflammation remains omnipresent. Research has demonstrated a positive effect on the modulation of OA symptoms through diet and exercise to promote an anti-inflammatory environment. More specifically, omega-3 PUFAs have demonstrated a reduction in inflammatory biomarkers and cartilage degradation, counteracting the natural disease state of OA. In addition to their chondroprotective role, omega-3 supplementation has been shown to have indirect positive effects on muscle tissue recovery following exercise, which is necessary to prevent the progression of OA and maintain an independent, healthy lifestyle. The effects of omega-3 supplementation on the disease state of OA and its symptoms remain inconclusive. Further clinical trials utilizing human participants are warranted to provide a conclusive recommendation on standardized supplementation of omega-3 for the modulation of osteoarthritis.

## Figures and Tables

**Table 1:** Radiological features of osteoarthritis.

Grade	Classification
0	No evidence of OA, normal radiographic findings
1	Doubtful narrowing of the joint space with possible osteophyte formation
2	Possible narrowing of joint space with definite osteophyte formation
3	Definite narrowing of joint space, moderate osteophyte formation, some sclerosis, and possible deformity of bony ends
4	Large osteophyte formation, severe narrowing of the joint space, with marked sclerosis and definite deformity of bone ends
